# Custom Ocular Prosthesis: A Case Report

**DOI:** 10.7759/cureus.41176

**Published:** 2023-06-30

**Authors:** Shreya Colvenkar, Shetti Thushara, Gona Maheshwar Reddy, Siliveri Shamili, Linju Vijay

**Affiliations:** 1 Department of Prosthodontics, MNR Dental College and Hospital, Sangareddy, IND; 2 Department of Prosthodontics, Sri Sai College of Dental Surgery, Hyderabad, IND; 3 Department of Conservative Dentistry and Endodontics, Sri Balaji Dental College and Hospital, Hyderabad, IND

**Keywords:** ocular, prosthesis, eye, photograph, digital, custom

## Abstract

Ocular prostheses play a vital role in restoring the appearance and functionality of the eye for individuals who have suffered from eye loss due to trauma, surgery, tumors, or congenital eye defects. This case report presents the successful fabrication and fitting of a custom-made ocular prosthesis for a patient with an eye defect. The process involved careful examination and digital imaging for the fabrication of prostheses. The custom prosthesis provided an excellent aesthetic match, improved comfort, and enhanced the patient's self-confidence, ultimately leading to an improved quality of life.

## Introduction

The human eye is an incredible organ that gives us the ability to make sense of our environment. The loss of a human eye has far-reaching implications for a person’s mental, physical, and social well-being [1]. It can be caused by a variety of reasons, including congenital defects, irreparable trauma, tumors, and sympathetic ophthalmia [2]. Surgical procedures to remove eyes can be broadly divided into three categories: evisceration, enucleation, and exenteration [2].

Ocular prostheses offer a viable solution by restoring the natural appearance of the eye, providing a sense of normalcy, and improving self-esteem and quality of life [3]. This type of prosthesis comes in two categories: stock and custom-made. Ready-made prostheses come in standard sizes, shapes, and colors, while custom-made prostheses offer a superior fit, even pressure distribution, superior eyelid movement, improved aesthetics, and enhanced functionality [4-10].

Ocular prostheses are typically made from biocompatible materials such as medical-grade acrylics or silicone elastomers [7,10]. These materials are lightweight, durable, and resistant to moisture and bacteria. Fabrication techniques have also evolved, with computer-aided design and three-dimensionally enabling the precise and efficient production of custom ocular prostheses. This article describes a simple, cost-friendly technique to fabricate acrylic ocular prostheses using digital photography.

## Case presentation

**Figure 1:**
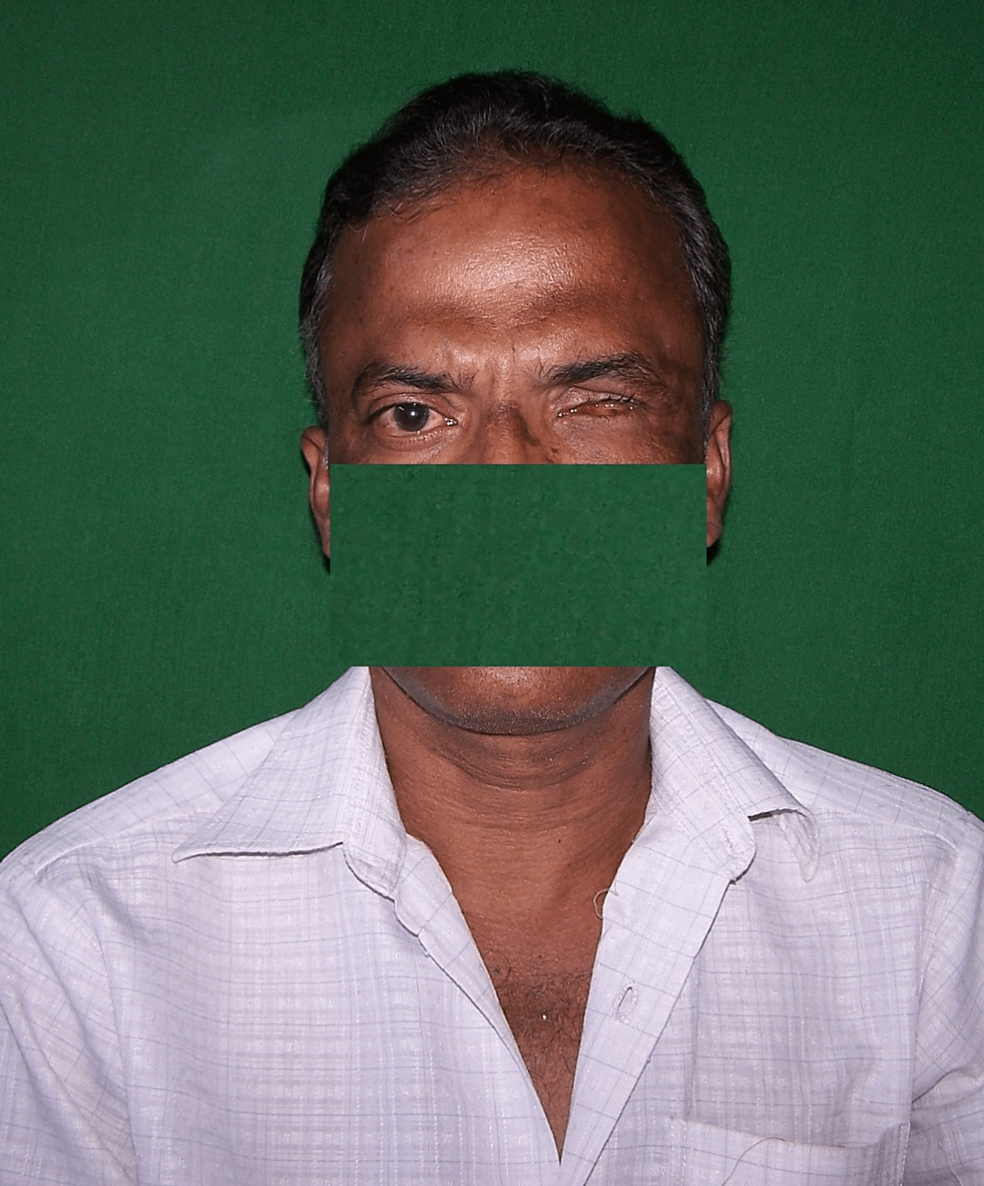
Pre-treatment

A male patient, 52 years old, presented to the prosthodontics department for the fabrication of an ocular prosthesis. History revealed that two years prior, he had experienced an injury to his left eye, which required surgical enucleation (Figure 1).

On examination, the socket showed no signs of inflammation and had sufficient depth and movement between the upper and lower eyelids. After carefully weighing all available treatment alternatives and discussing them with the patient, the decision was reached to create a custom acrylic ocular prosthesis that would fit the patient comfortably and appear as natural as the patient's other eye.

**Figure 2:**
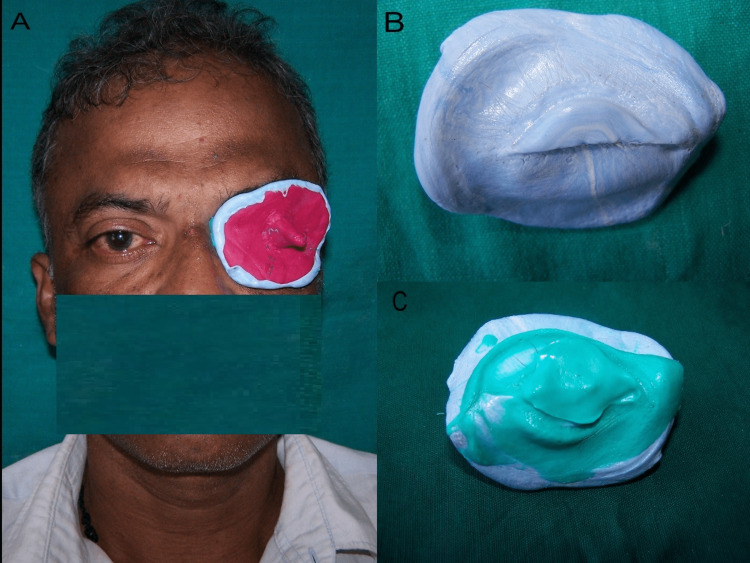
(A) Impression procedure, (B) impression with putty, and (C) final impression with light body using functional movement

The patient's eye was first irrigated with saline water, and a thin layer of vaseline was applied. An ocular tray with a handle was made using an impression compound. The patient was asked to close the eye, and the impression compound was adapted over the outer surface of the eye. To give the putty impression material mechanical retention, serrations were made into the impression compound tray. The socket impression was made using a putty silicone impression material to record the overall extent of the defect, followed by a light body wash impression material. The patient was instructed to maintain a straight gaze at a faraway object during the impression procedure (Figure 2).

**Figure 3:**
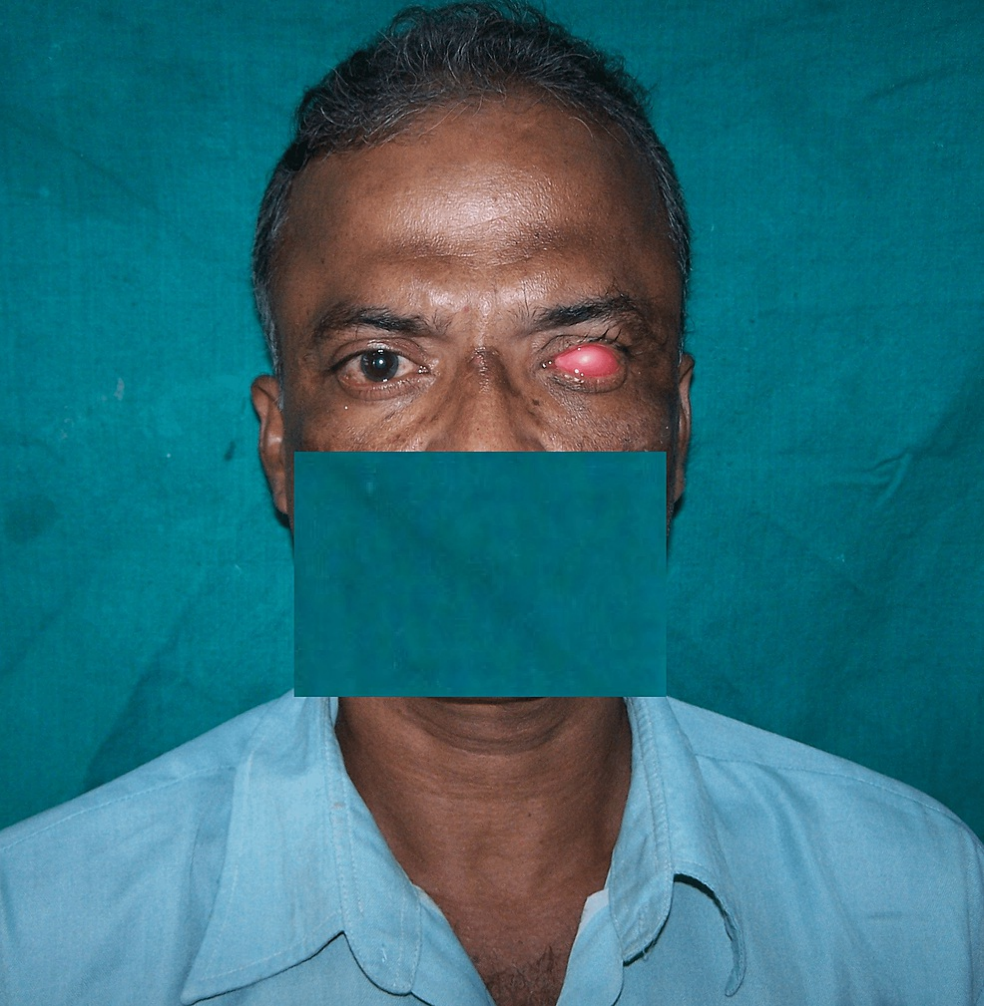
Wax conformer try-in

The impression was boxed and poured in type III dental stone using a two-pour cast technique. To create a wax conformer, molten modeling wax was poured into the master cast after it had been lubricated with a separating medium. The wax conformer was smoothed out and placed in the patient's enucleated socket. The retention of the wax conformer was checked by asking the patient to make all eye movements (Figure 3).

The iris of the contralateral natural eye was measured with a measuring ruler, and it was found to be 12 mm in size. A digital single-lens reflex (DSLR) camera was used to capture a digital image of the patient's contralateral natural eye. The iris image with different resolutions and contrast measuring 11 mm was printed on a photo gloss self-adhesive paper.

**Figure 4:**
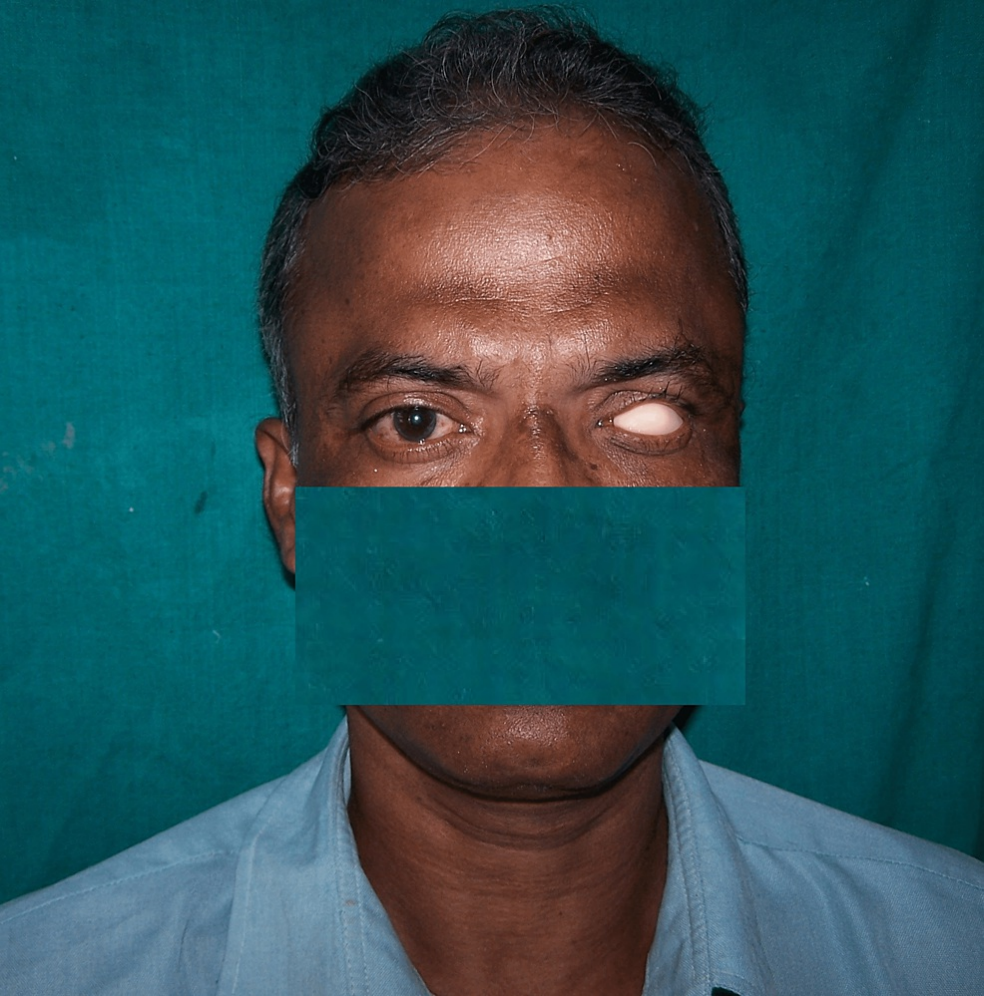
Acrylic sclera try-in

The wax conformer was acrylised according to the manufacturer’s instructions using the conventional method with clear heat-cured acrylic resin to obtain a scleral blank. The scleral blank was tried in the patient’s eye, and retention was verified by asking the patient to do all eye movements (Figure 4).

**Figure 5:**
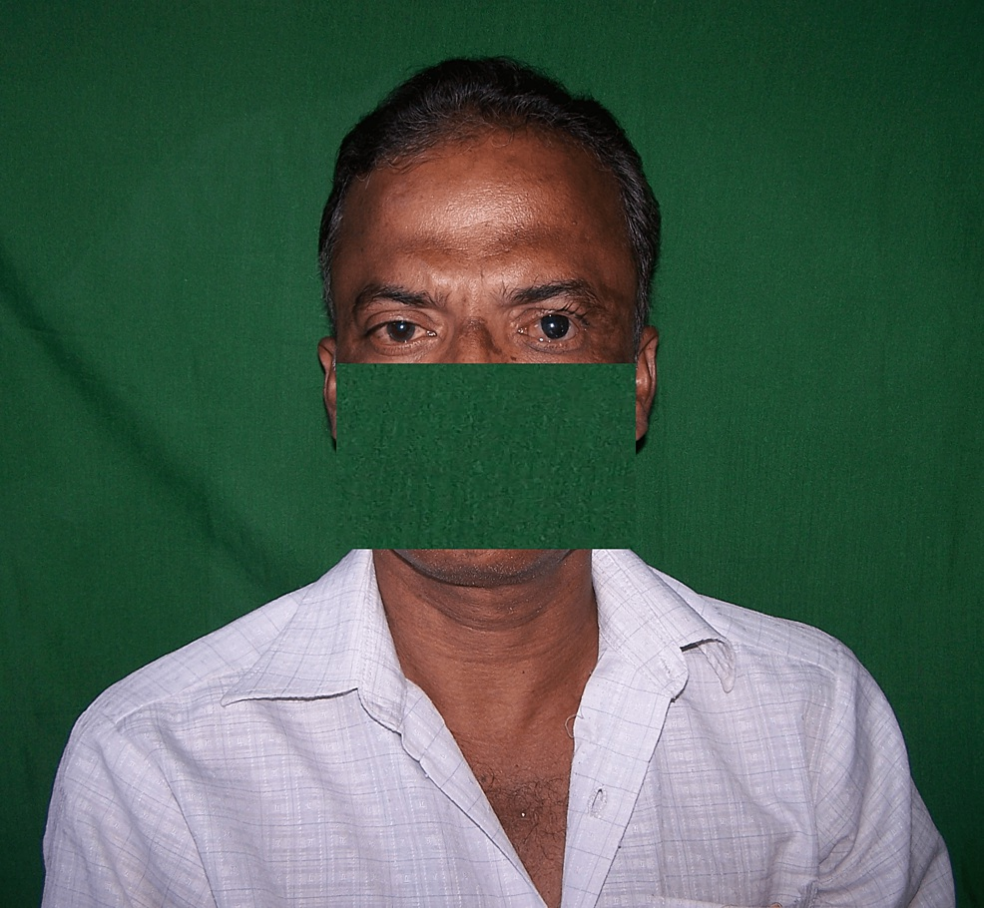
Post-treatment

On the entire frontal surface area, the scleral blank was smaller by 1 mm. The best glossy sticker of the iris was matched with the natural eye. The iris sticker was covered with cellophane to preserve the ink. The graph grid method was used to transfer the iris onto the scleral blank. The iris was then pasted onto the scleral blank. This was followed by the application of blood capillaries and the final coloration of the sclera. The entire sclera was covered with a 1 mm thickness of modeling wax. This wax would then be replaced by transparent heat-cured acrylic resin. This was followed by curing using a conventional technique according to the manufacturer’s instructions. The ocular prosthesis was polished and finished while taking care to maintain its convexity and contour. After being cleaned and lubricated with an ocular lubricant to maintain a tear film over the prosthesis and to assist eye movements, the prosthesis was placed in the socket (Figure 5).

After instructing the patient on how to take care of and maintain the prosthesis, the ocular prosthesis was delivered to the patient.

## Discussion

The eye, with its complex structure and vital role in visual perception, plays a crucial part in our daily lives. Individuals who experience eye loss due to various reasons face not only physical but also psychological challenges [2]. Ocular prosthesis fabrication is a complex process that requires a combination of technical and artistic skills. Various techniques have been described in the literature to make the fabrication process easier and more efficient. A custom tray fabrication for ocular impressions has been advised by a number of authors [3-10]. The use of a relining material to adapt an existing stock or custom ocular prosthesis to achieve an acceptable fit has also been suggested [6]. The custom tray fabrication in this case was simple and quick.

Numerous authors have extensively used polymethyl methacrylate (PMMA)-based ocular prostheses [7]. The incorporation of porcelain sclera veneer has also been suggested, which is costly and time-consuming [9]. Medical-grade silicone has been suggested because of its flexibility and reduced weight compared to acrylic [10]. In the present case, acrylic was used for strength, translucency, and cost.

The iris can either be painted or a stock iris can be added to match the patient’s adjacent eye [7]. In the present case, digital photography was used to duplicate the iris. Adobe Photoshop was used to edit the image to be printed on self-adhesive glossy photo paper. An iris picture was 1 mm smaller in diameter than the measured contralateral side to compensate for the magnification caused by the acrylic layer. The sclera was oil painted to match the contralateral eye. Research studies have concluded that oil paints have greater resistance to aging.

Kale et al. used digital photography to fabricate the sclera and iris and vacuum-pressed a clear co-polyester sheet onto the photo paper [8]. The technique, despite being difficult and time-consuming to master, successfully mimicked the patient's natural eye.

The method described in the present case was reasonably simple, affordable, and effective. Due to the exact replication of the patient's contralateral natural eye with an iris photograph and the painting of the sclera with oil paints, the clinician had a predictable outcome.

## Conclusions

Custom ocular prosthesis fabrication is a complex process that requires precision and accuracy. However, with the help of digital photography, this process can be simplified and made more efficient. This article describes a novel technique for custom ocular prosthesis fabrication using digital photography that can save time and effort for clinicians. This technique provided a comfortable and secure fit for the wearer while still maintaining a natural-looking appearance.
